# Gastrointestinal Perforation Complicated by Continuous Opioids Administration for Intrahepatic Cystic Hemorrhage Pain Management

**DOI:** 10.7759/cureus.27829

**Published:** 2022-08-09

**Authors:** Ayaka Matsuoka, Hiroyuki Koami, Taku Goto, Kota Shinada, Yuichiro Sakamoto

**Affiliations:** 1 Emergency and Critical Care Medicine, Saga University Hospital, Saga, JPN; 2 Emergency Medicine, Takagi Hospital, Fukuoka, JPN

**Keywords:** abdominal pain, lower gastrointestinal tract perforation, chronic renal failure, intrahepatic hemorrhage, multiple hepatic cysts

## Abstract

Intracystic hemorrhage is a rare complication of multiple hepatic cysts and can lead to hemorrhagic shock. Hence, measures should be taken to prevent the rupture of cysts. The incidence of intestinal perforation is high in patients undergoing hemodialysis. The diagnosis can be difficult in a patient without typical symptoms. We report the case of a woman in her late 60s with multiple renal and hepatic cysts, which caused chronic renal failure managed with dialysis. She presented with abdominal pain and was diagnosed with intrahepatic cystic bleeding. Continuous intravenous fentanyl was administered for pain management, which temporarily alleviated pain, but abdominal pain recurred with increased intensity when she resumed feeding. Subsequently, a contrast-enhanced computed tomography revealed perforation of the lower gastrointestinal tract. Therefore, in cases of intrahepatic cystic hemorrhage that require administration of analgesics, the complications of other diseases that may also cause acute abdominal pain should also be considered.

## Introduction

Intrahepatic cyst hemorrhage is a relatively rare complication with multiple liver cysts, with a reported incidence of 2-5% [1]. There have been reports of intrahepatic hemorrhage leading to rupture of hepatic cysts, resulting in hemorrhagic shock [1]. When treating hepatic cystic hemorrhage conservatively, measures should be taken to prevent cyst rupture [2]. Gastrointestinal disorders are common in patients undergoing hemodialysis due to various factors, including hemodialysis-induced circulatory failure, autonomic neuropathy, immune dysfunction, amyloidosis, and hemostatic dysfunction [3]. Patients undergoing hemodialysis also have a higher incidence of acute abdominal disorders, including perforation of the lower gastrointestinal tract [4]. However, diagnosis of acute abdominal disorders in patients undergoing hemodialysis is complicated as the abdominal pain may present with non-specific symptoms and is not accompanied by an elevated white blood cell (WBC) count [5,6].

In the present study, we report a case wherein the diagnosis of lower gastrointestinal perforation in a patient on maintenance hemodialysis was complicated by the use of continuous intravenous fentanyl as an analgesic for pain caused by intrahepatic cystic hemorrhage.

## Case presentation

The patient was a woman in her late 60s with a history of polycystic liver diseases (PLD) associated with autosomal dominant polycystic kidney disease (ADPKD), resulting in chronic renal failure. The patient was on maintenance hemodialysis for seven years before being admitted to our hospital. She had also undergone stent-graft insertion for a left common iliac artery aneurysm. Her paroxysmal atrial fibrillation was managed with warfarin for prevention of thrombosis, and she was not on any other anticoagulants or steroid therapy. Other comorbidities included valvular heart disease, hypertension, dyslipidemia, and hyperuricemia.

On the day of admission, the patient presented with upper abdominal pain that worsened on inhalation since she woke up. The feces were brown and soft. Maintenance dialysis was started by her family doctor. However, she was referred to our hospital owing to increasing upper abdominal pain and nausea. Her height and weight were 155 cm and 46 kg, respectively. Respiratory rate, SpO_2_, blood pressure, and pulse rate were 15 breaths/min, 94%, 162/92 mmHg, and 97 beats/min, respectively, and she remained conscious. Pallor on the eyelid conjunctiva and yellowing of the ocular conjunctiva were absent. Abdominal tenderness was present and mainly localized to the left side of the upper abdomen. Additionally, localized muscular defense and diminished intestinal peristalsis were observed.

On presentation, blood tests showed a WBC count of 2,900/µL, a hemoglobin level of 11.9 g/dL, prolonged prothrombin time test-international normalized ratio of 1.56, elevated D-dimer of 6.18 µg/mL, and c-reactive protein (CRP) level of 1.74 mg/dL. Venous blood gas findings (room air) were pH of 7.282 and lactate of 3.3 mmol/L.

**Figure 1:**
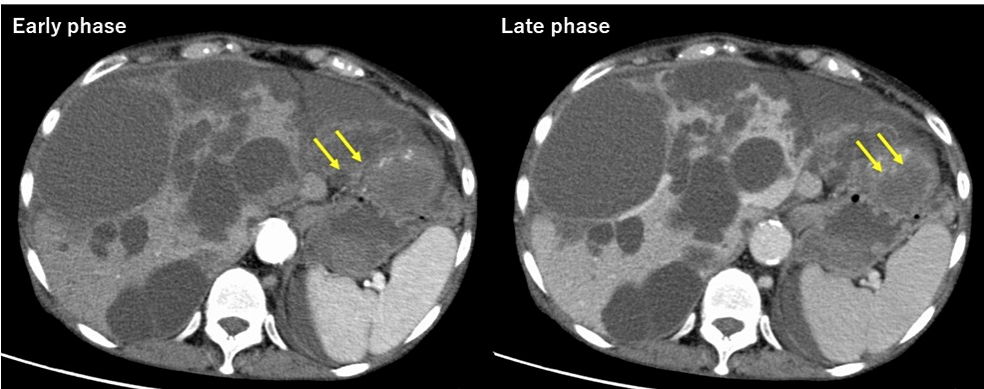
Contrast-enhanced CT scan of the abdomen on admission Contrast-enhanced CT shows multiple liver cysts. In the early contrast-enhanced phase, a strong enhancing effect is observed in cysts in the lateral zone of the left lobe of the liver. In this area in the late contrast phase, the enhancing effect is diffused, suggesting intracystic hemorrhage.

Abdominal contrast-enhanced computed tomography (CT) scan showed multiple cysts throughout the liver. The mass in the lateral segment of the left lobe showed heterogeneous enhancement in the early contrast phase and diffused enhancement in the late phase, indicating intrahepatic cystic hemorrhage. Edematous changes were also observed from the rectal to the sigmoid colon wall, suggesting coexisting enterocolitis. Although the left common iliac artery aneurysm did not change in size, an endoleak was noted (Figure 1).

The patient was diagnosed with intrahepatic cystic hemorrhage and admitted to the ICU with a conservative treatment plan due to the absence of anemia progression and stable hemodynamic status.

On admission, there were no significant changes in vital signs. Since the patient’s abdominal pain was severe with a numerical rating score (NRS) of 7, fentanyl (0.05 mg/h) was administered continuously as an analgesic. Since the CT scan indicated enteritis, the patient was put on fasting till the abdominal pain was reduced.

**Figure 2:**
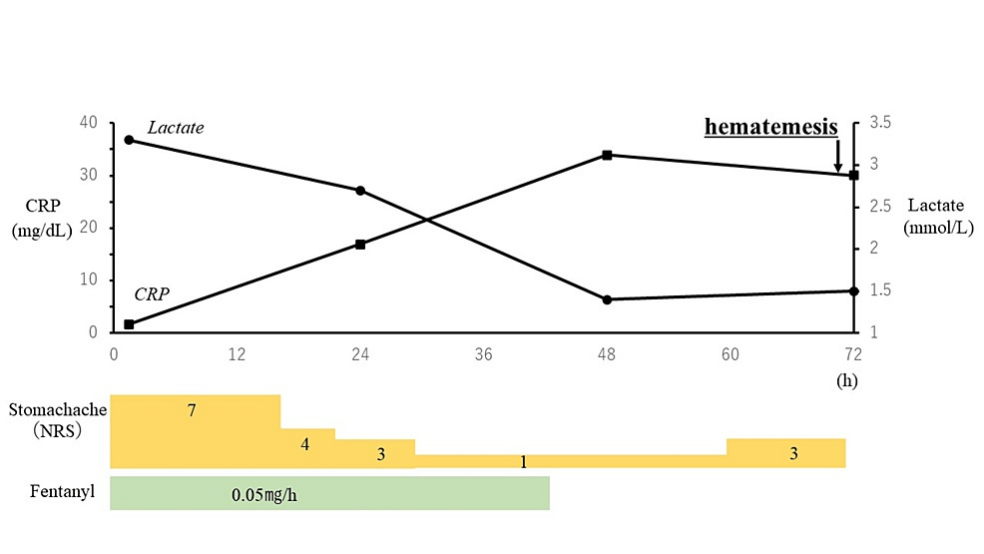
Clinical course post-hospitalization Graphical representation of the levels of CRP (mg/dL) and lactate (mmol/L) up to 72 hours after hospitalization. Abdominal pain represented using the NRS is shown here using a bar graph. The duration of continuous fentanyl administration is shown in green. CRP: c-reactive protein; NRS: numerical rating score

After fentanyl administration was started, abdominal pain had reduced, and 24 hours after admission to the ICU, the abdominal pain had almost disappeared with an NRS of 1. Hence, continuous intravenous fentanyl administration could be discontinued. Abdominal echocardiography showed no increase in fluid retention of the hepatic cysts over time. Blood tests showed no decrease in hemoglobin level and stable circulation dynamics. CRP was elevated (16.9 mg/dL), but fever and elevated WBC count were not observed. After confirming that there was no increase in abdominal pain and no anemia progression, the patient was started on oral liquid nutrition 36 hours after admission to the ICU, and abdominal pain increased again. However, the site of abdominal pain was not consistent, and there were no symptoms of peritoneal irritation. The patient subsequently developed fever, became restless, and complained of pain in the entire abdomen (NRS 3). There was no evidence of board stiffness or recurrent pain. Seventy hours after admission to the ICU, a large amount of dark red blood was observed in the feces. Blood test findings showed an elevated CRP of 33 mg/dL (Figure 2).

**Figure 3:**
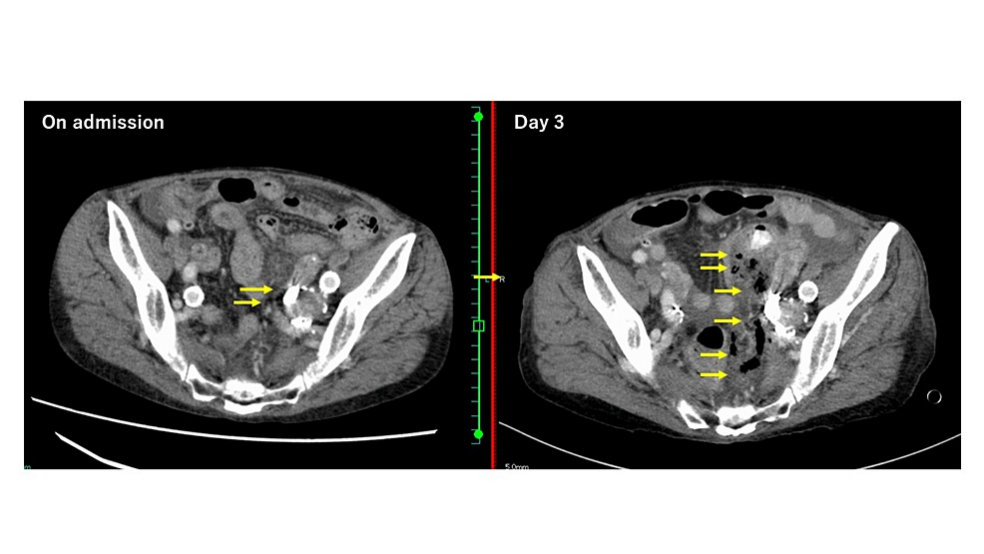
Comparison of abdominal contrast-enhanced CT scan on admission with that on day 3 Images that were taken on admission show a small amount of free air in the mesentery. The day 3 image shows a discontinuity in the sigmoid colon wall and free air and bowel contents in the surrounding mesentery.

Contrast-enhanced CT scan revealed the cause of the increased abdominal pain and bloody stools. There was a discontinuity in the sigmoid colon and a fecal mass and free air in the mesentery (Figure 3; right). Retrospectively, contrast-enhanced CT at the time of initial treatment showed an edematous and thickened colon wall, discontinuity in a small area of the sigmoid colon wall, and a minute amount of free air in the mesentery (Figure 3; left).

However, there was no change in the size or increase in hematoma of the hemorrhagic liver cyst. Thus, the patient was diagnosed with a perforation of the sigmoid colon, and emergency surgery was deemed necessary. Since our operating room was full, the patient was transferred to an available facility. The emergency surgery was performed on the same day. Intraoperative findings showed perforation of the sigmoid colon diverticulum and the formation of a retroperitoneal abscess. Abdominal cavity drainage and Hartmann surgery were performed.

Postoperatively, the patient had a stent-graft infection of the left common iliac artery, which required long-term antimicrobial treatment, but her condition improved after the surgery, and she was transferred to a rehabilitation facility on the 53rd day.

## Discussion

In the present case, a small amount of free air was missed on the initial CT, and after admission to the ICU, the symptoms of lower gastrointestinal perforation were masked by continuous analgesia. This delayed the diagnosis of lower gastrointestinal perforation. In gastrointestinal perforation, detection of free air on abdominal CT has been reported to be most conducive to diagnosis. On the other hand, the diagnostic sensitivity of free air for lower gastrointestinal perforation has been reported to be 70-80% [7]. In this case, the diagnosis of hepatic cyst hemorrhage was made on the initial CT and this was considered the cause of the abdominal pain, which may have led to missing the very small amount of free air present. The possibility that small amounts of free air may be difficult to diagnose should be considered.

PLD is relatively rare and may be present alone or in association with ADPKD and autosomal recessive polycystic kidney disease [8,9]. Approximately 80% of patients with PLD are asymptomatic, while the remaining 20% develop complications such as organ compression symptoms, hemorrhage, and infection due to liver cysts [8]. Risk factors for the progressive growth of liver cysts in patients with PLD include older age, sex, exposure to excess estrogen, and severe renal dysfunction [7].

Intrahepatic hemorrhage is a rare complication of hepatic cysts [2], with a reported incidence of 2-5% [10]. Intrahepatic cystic hemorrhage is caused by increased internal or external pressure, resulting in necrosis of the cyst wall or detachment of small blood vessels [11]. Cyst hemorrhage and rupture are rare, but cases leading to hemorrhagic shock have been reported [10]. When rupture is accompanied by hemodynamic instability, vascular embolization or surgical resection is required [12]. In this case, contrast-enhanced CT showed extravascular leakage, but conservative treatment was chosen because there was no evidence of cyst rupture, hemodynamic instability, or advanced anemia.

Intrahepatic cystic hemorrhage often presents with sudden right upper quadrant abdominal pain, and sudden abdominal pain has been reported in 85% of the cases [1,13,14]. Intracystic hemorrhage without rupture is generally less severe, with pain often worsening early in the course of the disease and then becoming less severe [1]. However, Amanda et al. reported a case of intrahepatic cystic hemorrhage with severe abdominal pain that was resistant to various analgesics [2]. Moreover, the patient complained of severe upper abdominal pain from the time of arrival at the hospital to the extent that she could not remain still. Prevention of cyst rupture is important in the acute management of hepatic cyst hemorrhage [2]. Considering the risk of hemorrhagic shock on rebleeding or cyst rupture and the possibility of requiring vascular embolization or surgical intervention, we considered that strict pain management was necessary, and fentanyl was started as an analgesic.

The patient’s abdominal pain was confined to the upper abdomen on the first day but flared up on the third day and involved the entire abdomen. This indicated that the cause of the abdominal pain had shifted from a hepatic cyst hemorrhage to generalized peritonitis due to perforation of the lower gastrointestinal tract. However, the abdominal pain due to lower gastrointestinal perforation and peritonitis may have been masked by the continuous intravenous fentanyl administration. The dose of fentanyl used at the start was 0.05 mg/h, but it may have been necessary to start with a smaller dose or to use a protocol of finely calibrated increases or decreases according to abdominal pain.

Perforation of the gastrointestinal tract is a severe and surgical emergency [15,16]. The incidence of acute abdominal disorders, including gastrointestinal perforation, has been reported to be 2.1% higher in patients undergoing long-term hemodialysis than in patients not undergoing dialysis [3,4,17]. This is thought to be due to amyloidosis, atherosclerosis, vascular calcification, hemodynamic fluctuations, and constipation in patients on hemodialysis, [3]. In patients undergoing hemodialysis, compromised immunity, absence of leukemia, and typical abdominal pain symptoms complicate the diagnosis of acute abdominal disorders [5,6,18].

Diverticular perforation is reported to be the most common cause of lower gastrointestinal perforation in long-term maintenance hemodialysis patients [19]. It is also known that the longer the duration of hemodialysis, the higher the risk of gastrointestinal perforation [3]. This patient had been on maintenance hemodialysis for seven years and was at high risk of acute abdomen.

In the present case, abdominal pain was confined to the upper abdomen on the first day, and there was no elevated inflammatory response, including elevation of leukocyte counts. The patient’s initial diagnosis of intrahepatic cystic hemorrhage causing upper abdominal pain complicated the diagnosis of lower gastrointestinal perforation.

Acute abdominal disorders in patients undergoing dialysis are characterized by elevated CRP rather than elevated leukocytes, and in this case, CRP was also elevated over time during hospitalization [3]. We believe that this CRP elevation and the migration of abdominal pain from the upper quadrant on the first day to the entire abdomen on the third day indicated lower gastrointestinal perforation.

## Conclusions

We reported a case wherein the diagnosis of lower gastrointestinal perforation was complicated by intrahepatic cystic bleeding. The intrahepatic cystic bleeding presented as abdominal pain and resulted in the use of fentanyl for analgesia. Intrahepatic cyst bleeding is a relatively rare complication of multiple hepatic cysts and rarely causes abdominal pain severe enough to warrant intervention with narcotic analgesics. In cases of intrahepatic cystic hemorrhage that require administration of analgesics, including this case, it is necessary to note the complications of other diseases that may also cause acute abdominal pain.
